# Clinical evaluation of marginal fit of uncemented CAD-CAM monolithic zirconia three-unit restorations in anterior areas, using scannable and conventional polyvinyl siloxane impression materials

**DOI:** 10.1186/s12903-023-02771-z

**Published:** 2023-01-30

**Authors:** Mohammad Hassan Kalantari, Benika Abbasi, Rashin Giti, Zahra Rastegar, Saeid Tavanafar, Sheila Shahsavari-pour

**Affiliations:** 1 Department of Prosthodontics, School of Dentistry, Shiraz University of Medical Science, Shiraz, Iran; 2 Department of Prosthodontics, School of Dentistry, Isfahan University of Medical Science, Isfahan, Iran; 3 Department of Periodontology, School of Dentistry, Shiraz University of Medical Science, Shiraz, Iran; 4 Department of Oral and Maxillofacial Surgery, School of Dentistry, Birjand University of Medical Sciences, Birjand, Iran; 5 Department of Oral and Maxillofacial Surgery, School of Dentistry, Shiraz University of Medical Science, Shiraz, Iran

**Keywords:** Marginal fit, Monolithic zirconia, FPD, Scannable impression materials–conventional impression material

## Abstract

**Background:**

The accuracy of impression techniques determines the marginal fit of fixed prostheses. Marginal accuracy plays a main role in the success and failure of treatments. This in-vivo study evaluated the marginal fit of anterior three-unit monolithic zirconia fixed partial dentures (FPDs) using conventional and scannable polyvinyl siloxane impression materials.

**Methods:**

Ten patients were selected to replace the lateral teeth with a three-unit monolithic zirconia bridge. For each patient, in the first group, an impression was made with a two-step putty-wash technique using scannable polyvinyl siloxane material (BONASCAN; DMP, Greece). In the identical session, as the second group, an impression of conventional putty-wash polyvinyl siloxane was taken (BONASIL A^+^ Putty; DMP, Greece). The marginal discrepancy was measured through the replicas, which were cut perpendicularly within the buccolingual and mesiodistal directions. An Independent t-test was employed for data analyses (*P* < 0.05).

**Results:**

The marginal discrepancy in a conventional method for central abutment in mid-buccal, mid-lingual, mid-mesial, and mid-distal was higher than in the scannable method but it was not significant (*P* > 0.05). Also, the marginal discrepancy for canine abutment in the conventional method was higher than in the scannable method, but it was not significant, either (*P* > 0.05).

**Conclusions:**

FPDs fabricated from both scannable and conventional impression materials were not superior to each other in marginal fit for both central and canine abutments by evaluation using the replica technique.

## Background

An essential phase in prosthesis construction is the molding operation, which precisely transfers a patient's soft and hard tissues to the laboratory. The accuracy of impression techniques determines marginal fit, which is the primary purpose of any prosthetic treatment [1–3]. The feature of impression materials, not deforming during the setting time, expresses dimensional accuracy. Polyvinylsiloxane, also known as additional silicone, has the least amount of permanent deformation [2]. Two common molding techniques can be mentioned: (1) one-step putty-wash and (2) two-step putty-wash. Putty acts as a tray for light body, and wash can record details because of its good flow [4].

After the introduction of glass ceramics, ceramic materials have been widely used for several decades [5] because of high esthetic demands, chemical stability [6], high biocompatibility, and technological developments [7, 8]. Polycrystalline ceramics such as Aluminum Oxide (Al_2_O_3_) and Zirconium dioxide (Zirconia, ZrO_2_), due to their relatively lower cost, are used more than other ceramics [9]. Several oxides are added to zirconia to stabilize the tetragonal and/or cubic phases. Among other oxides (MgO, CeO,CaO) which are added to pure zirconia, yttrium oxide (Y_2_O_3_) is the most common stabilizing oxide, added at a rate of 2–5 molar percentage [10]. It has been decades since the presence of dental computer-aided design (CAD) and computer-aided manufacturing (CAM) in dentistry; a straightforward approach and also the speed of construction can be delineated [11, 12]. Many studies have shown that a digital impression is more acceptable than conventional methods, with recent advances in scanners and increased accuracy and their ability for three-dimensional measurement. This is because of the preference of the dentist and patient and the time that it takes [13]. Two different intraoral and extraoral scanning methods are used to achieve digital models. New intraoral scanning techniques using Laser and LED technology create a 3D quality image of intraoral structures, yet rotating bases are applied in extraoral scanners, which use 3D laser surface technology [14]. These two methods have advantages and disadvantages. Factors such as inadequate access, saliva, and movements during intraoral scanning create errors during digitization. Also, extraoral scanning will affect the prosthesis fit because of deformations such as impression material shrinkage or air bubbles and impacts the accuracy of the stone cast, including an expansion or increased falsity because of using powder to reduce reflection [15]. Instead, with the development of scannable elastomeric impressions, we can scan them without pouring a stone cast. The difference between scannable elastomeric impressions and conventional ones is related to their color, physical characteristics, and brightness of surface material, which are hypothesized to enhance digitization [16].

Among three factors, marginal fit, fracture resistance, and esthetics, considered for the success of ceramic restorations [17], the marginal fit is directly related to impression accuracy. Secondary caries at the crown margin and the inflammation of periodontal tissues may occur because of oral fluids and chemo-mechanical forces owing to sizable marginal discrepancies [18]. A critical measurement for evaluating prosthesis fit is discrepancies between the marginal of abutment teeth and retainers after the prosthesis is seated on the teeth. Molin and Karlson first described the technique used to measure the gap space as the replica technique [15]. Despite the disadvantages of the replica technique, most studies considered it as a reliable method to evaluate the marginal and internal discrepancies [16, 17].

There is an intensive relationship between the accuracy of impression techniques and the marginal fit, it can be also stated that marginal accuracy plays a key role in the success and failure of prosthesis treatments. Not enough studies have addressed the accuracy of scannable impression materials. Hence, this in-vivo study aimed at evaluating the marginal fit of three-unit monolithic zirconia FPDs using conventional and scannable polyvinyl siloxane impression materials. The null hypothesis was that there is no difference between conventional and scannable polyvinyl siloxane impression materials on the marginal fit of monolithic zirconia accuracy.

## Methods

### Selection and tooth preparation

The Ethical committee approved this study. Among patients referred to the prosthodontics department of the School of Dentistry, ten people were selected for lateral teeth replacement with a three-unit monolithic zirconia bridge. All participants were above 16 years of age. At the beginning of the session, all of the patients completed a written informed consent form. Each person's central and canine teeth were prepared with 2 mm occlusal reduction and 1 mm for axials. A sectioned putty index was used to visualize tooth preparation. A round-end taper diamond bur was utilized for 0.3–0.5 mm radial shoulder margin, the preferred finish line for all-ceramic crowns [19].

### Impression methods and scanning

**Fig. 1:**
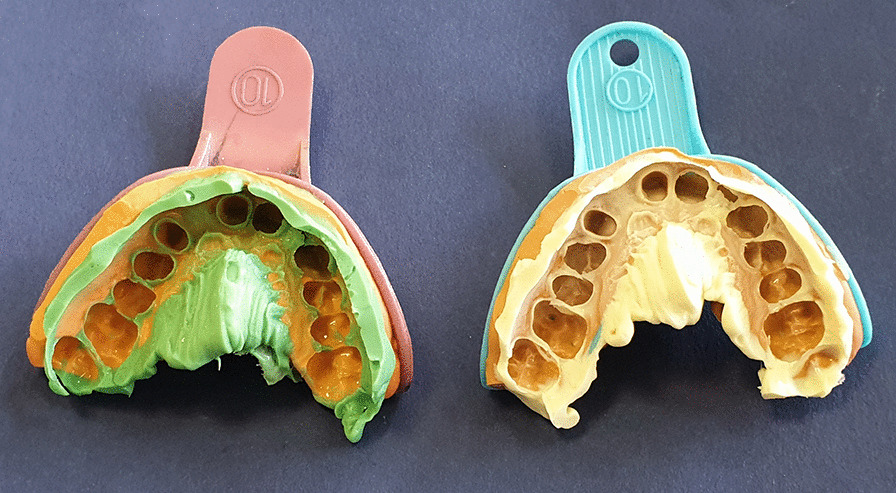
An impression of conventional putty-wash polyvinyl siloxane (left) and scannable putty-wash polyvinyl siloxane (right)

For each patient in the first group, an impression was taken with a two-step putty-wash technique using scannable polyvinyl siloxane material (BONASCAN; DMP, Greece). The resulting molds were SD laser-scanned (3Shape D810; 3Shape). Because of the properties of the scannable impression material, there was no need to use the spray while scanning the mold. In the identical session, out of ten patients, an impression of conventional putty-wash polyvinyl siloxane (BONASIL A^+^ Putty; DMP, Greece), as the second group, was taken with the same approach (Fig. 1). For ease of scanning the die surface, the second group’s cast was sprayed with scanning spray. The dies were also SD laser-scanned (3ShapeD810; 3Shape). The data obtained from both groups, mold scanned and die scanned, were converted into CAD data and designed in a computer software system (3Shape CAD Design software; 3Shape, Copenhagen, Denmark). Bridges were machined out of the multilayered system of monolithic zirconia (DD cubeX^2^®ML, Multilayer, Cubic Zirconia System, Dental Direkt, Germany) by the aid of a CAD-CAM machine (Cori Tec 340i; imes-icor GmbH, Eiterfeld, Germany). After milling, they were transferred to the furnace (ATRA, ATRA Factory) and sintered as per the manufacturer’s recommendations.

### Clinical evaluation

Then, within the clinical evaluation, one examiner adjusted the tissue surface of the pontics, proximal contacts, margins, occlusal, and contours for both three-unit bridges. Then a specialist checked them for final confirmation. Next, the retainers were filled with BONASCAN light body (BONASCAN; DMP, Greece) and were placed on the abutment teeth. The patients were asked to close their teeth on the cotton roll and keep close until the material was set entirely. This thin layer showed a mismatch between the inner surface of the retainer and the prepared teeth.

### Replica technique

**Fig. 2:**
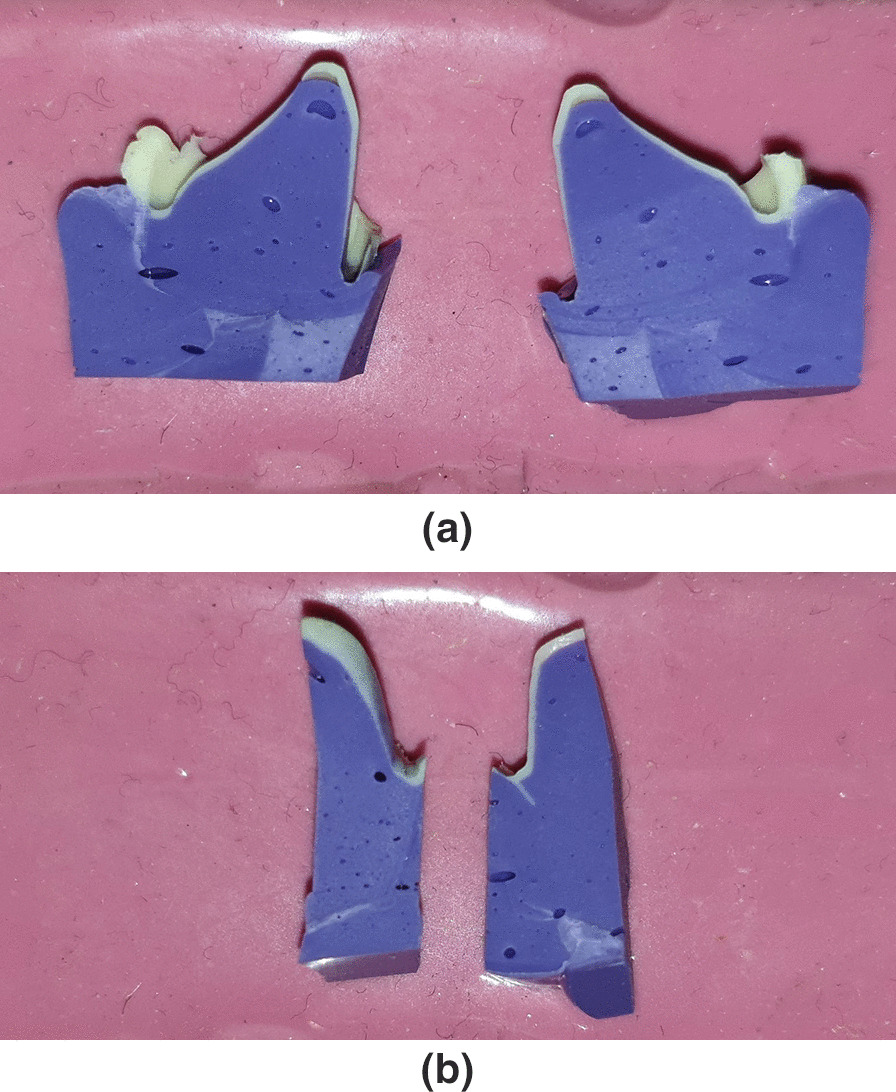
The replica was cut perpendicularly to the occlusal surface into four sections within the buccolingual and mesiodistal directions

Once setting the thin silicone layer, this layer was reinforced by injecting regular body silicone (Elite HD + ; Tray material Regular body-normal set, Zhermack, Italy). After the replica set, it was cut perpendicularly with a number 12 surgical blade to the occlusal surface. It was cut within the buccolingual and mesiodistal directions, in the middle points into four sections mid-buccal, mid-lingual, mid-mesial, and mid-distal. Then they were placed on clay or slab (Fig. 2).

### Measurement

The thickness of the outer silicone layer of the replica, in the middle of the finish line region, was measured with a microscope at 230× magnification (AM413FIT Dino-Lite Pro; Dino-Lite Electronic Corp) and an analysis software system (DinoCapture 2.0; AnMo Electronics Corp). The measurement software using the ruler tool showed marginal discrepancies. All the measurements were taken by the same operator. After measuring light-body silicone layer thickness, bridges with less average marginal discrepancies were cemented (GC Gold Label, radiopaque glass–ionomer luting and lining cement, GC Corporation, Tokyo, Japan).

### Statistical analysis

Data was analyzed using SPSS software, version 22.0 (IBM Corp, Armonk, NY, USA). Kolmogorov–Smirnov and Shapiro–Wilk tests showed that the data was normally distributed, and the variances were homogenous. An independent sample t-test was employed to compare the marginal fit of the two groups of conventional and scannable polyvinyl siloxane impression materials (α = 0.05).

## Results

**Fig. 3:**
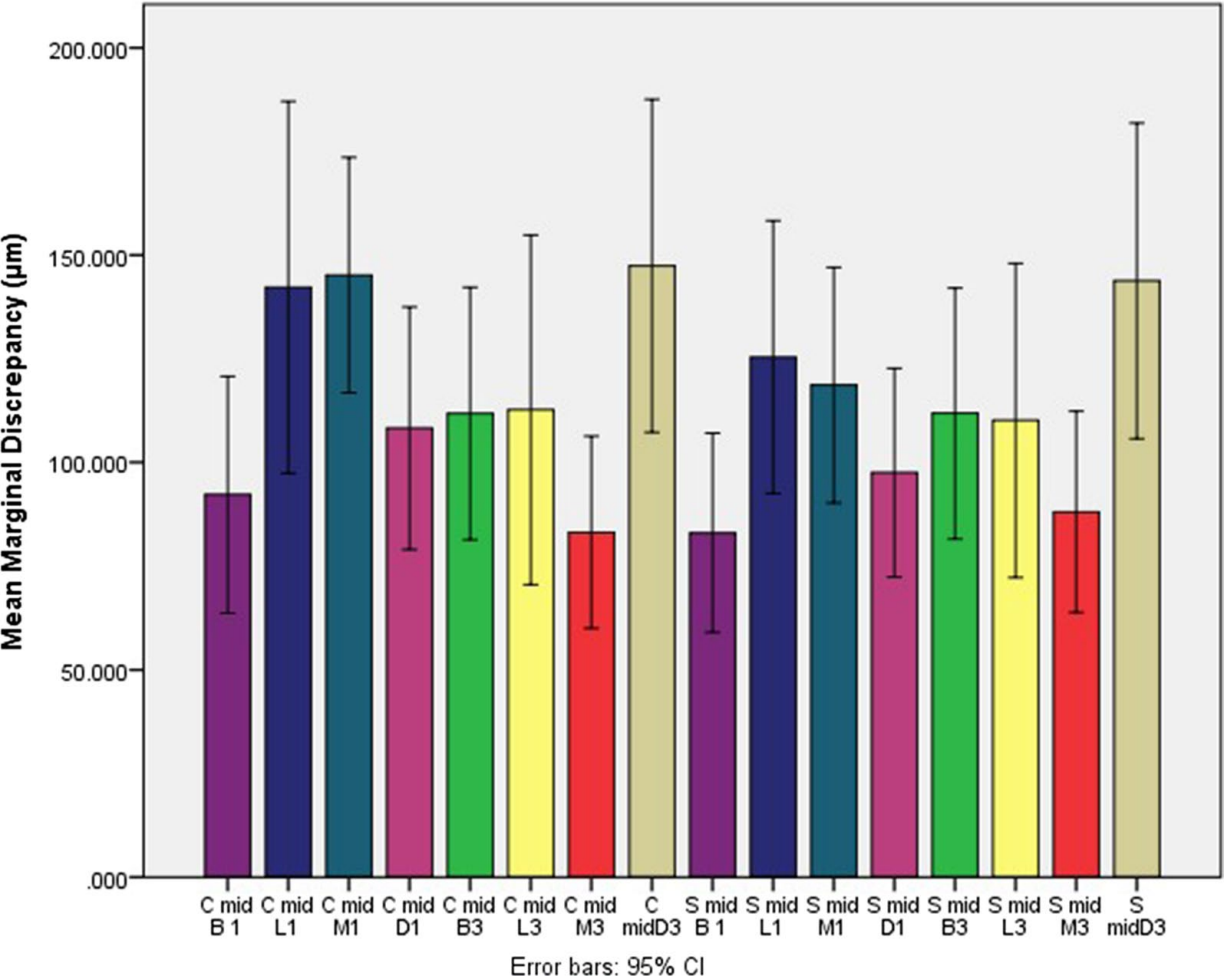
The mean and standard deviations of marginal discrepancy of the two impression methods. C: conventional method, S: Scannable method. B1: central mid buccal, L1: central mid lingual, M1: central mid mesial, D1: central mid distal, B3: canine mid buccal, L3: canine mid lingual, M3: canine mid mesial, D3: canine mid distal

The mean and standard deviations (SD) of marginal discrepancy between the two impression methods are shown in Table 1 and Fig. 3. The mean ± SD marginal discrepancy for both abutments in the conventional impression method in mid-buccal, mid-lingual, mid-mesial, and mid-distal was higher than in the scannable method. However, the marginal discrepancy in the conventional method compared with the scannable method in the central abutment in mid-buccal (*P* = 0.584), mid-lingual (*P* = 0.502), mid-mesial (*P* = 0.152), or mid-distal (*P* = 0.538) was not significant. Also, the marginal discrepancy in the conventional method compared with the scannable method in canine abutment was not significant either (*P* = 1.000, 0.921, 0.744, and 0.844 respectively).

**Table 1:** Mean ± standard deviation (SD) of discrepancy values (µm) in 4 middle points of each abutment

Abutment	Impression method	
	Mean ± SD	
	Conventional	Scannable
Central		
Mid-buccal	92.2 ± 39	83. ± 33.5
Mid-lingual	142.2 ± 62.8	125.4 ± 46
Mid-mesial	145.2 ± 39.8	118.6 ± 39.7
Mid-distal	108.2 ± 40.8	97.6 ± 35.2
Canine		
Mid-buccal	111.8 ± 42.5	111.8 ± 42.2
Mid-lingual	112.7 ± 58.9	110.2 ± 53
Mid-mesial	83.2 ± 32.4	88.1 ± 33.9
Mid-distal	147.4 ± 56.2	143.8 ± 53.2

## Discussion

This in-vivo study evaluated the marginal fit using conventional and scannable polyvinyl siloxane impression materials. The conventional and scannable polyvinyl siloxane impression materials did not significantly affect marginal fit accuracy.

Because scanning the incisal edges in molding materials and scanning methods are in great level of difficulty [20], the anterior teeth were selected for this study with the help of new impression scannable materials. According to the reports by Holmes and colleagues [21], and Wettstein and colleagues [22], the marginal fit is reflected by the marginal discrepancy. Based on several studies [23–25], the replica technique was used to assess the marginal fit to compare the marginal discrepancy between the conventional and scannable polyvinyl siloxane impression materials in the present study. In this study, that marginal discrepancy of both central and canine abutment teeth in the conventional impression method in the four mid-buccal, mid-lingual, mid-mesial, and mid-distal regions was not significantly different from marginal discrepancy obtained from the scannable impression material. Therefore, conventional and scannable polyvinyl siloxane impression materials would not significantly affect marginal fit accuracy.

Many studies assessed the marginal and internal fit of restorations made by digital impressions compared with conventional impressions using the replica technique, and comparable or even higher results were found for digital impression materials [26–31].

The gap could be a determinative issue for the long-term integration of a restoration. Although the maximum prosthetic precision for CAD-CAM restorations has not been stated clearly, the range of acceptable, marginal discrepancies was 50–120 µm. This gap can be detected clinically at the margin of a crown and result in multiple errors encountered throughout the crown fabrication step [25]. The replica technique is a reliable and accurate method for evaluating the accuracy of dental restorations, which also allows the quantification of discrepancies on inner surfaces and the marginal edge of the crown [23]. A relative disadvantage of the replica technique is the two-dimensional representation of results. However, compared to other techniques, most studies expressed that verifiable and accurate results were obtained through the replica technique [24, 25].

The morphology of elastomeric impressions facilitates photoconductivity of the IOSs or digitizing physical casts precisely in subgingival preparations and undercut or apical areas. Laboratory scanners cannot register the areas of surfaces beyond the incidence of the light they emit [16]. Besides the advantages mentioned for IOSs [32], factors such as saliva and patient movements that make errors during digitization can be factors for more inclination towards scannable elastomeric materials. In reviewing new elastomeric impression materials on the trueness and precision of dental casts obtained from the direct digitization, García-Martínez et al. [16] concluded that scannable impressions of two types of vinyl siloxanether impression materials using a laboratory laser scanner showed more accuracy than conventional elastomeric materials.

Several studies that compared the fitting accuracy of fixed prosthetics produced via digital and conventional impressions [15, 27, 28, 31, 33] showed that frameworks fabricated from digital impressions demonstrated better marginal fit than those fabricated from conventional impressions. In line with our results, several studies [26, 29, 30, 34] showed that crowns produced with intraoral scanning techniques offer a comparable or perhaps higher exactitude of marginal fitting accuracy.

In another study [35], the marginal accuracy of CAD/CAM restorations using different impression systems was compared as follows: group 1 (PVS impression scan), group 2 (stone cast scan), group 3 (Cadent iTero), and group 4 (Lava True Definition). With the aid of an optical comparator on each abutment, the marginal misfit of the zirconia FDPs was evaluated at four points. It was concluded that the marginal gap of all impression techniques was statistically significant and was within the acceptable clinical limit (120 μm).

Ahrberg and colleagues [27] evaluated the fit and efficiency of CAD/CAM-fabricated all-ceramic restorations using silicone replicas. Based on direct and indirect digitalization (a Lava C.O.S and a conventional polyether impression) concluded that a significantly better marginal fit was noted with direct digitalization. Another study [15] evaluating the marginal fit of CAD-CAM frameworks fabricated from an intraoral digital impression compared to conventional impression showed that intraoral digital impression systems were better. In another study [33], circumferential marginal gap measurements, including line angles, compare the marginal fit of crowns fabricated with digital and conventional methods. It was noted that the digital fabrication method provided a far better marginal fit than the conventional method. Furthermore, Syrek and colleagues [31] conducted an in-vivo study to analyze the marginal accuracy of a single ceramic crown based on a digital impression (Lava COS) and a conventional impression and showed that the digital group was significantly better than the conventional group.

Similar to our results, another study [34] showed that digital impression systems allowed the fabrication of fixed prosthetic restorations with similar accuracy as conventional impression methods. Moreover, Abdel-Azim and co-workers [26] used conventional impressions and two intraoral digital scanners to compare the marginal fit of lithium disilicate crowns fabricated with CAD/CAM technology. Both digital and conventional impressions were found to produce crowns with similar marginal accuracy. Also, Boeddinghaus and co-workers [29] concluded that compared with zirconia copings using four different techniques (three different intraoral digital and one conventional impression method), the marginal fit in intraoral and laboratory scans was equal. Also, Rödiger and colleagues [30], in a comparative clinical study of fitting accuracy of zirconia single crowns produced via digital and conventional impressions, noted that intraoral scanning techniques had the same or even higher marginal accuracy, which was consistent with our result.

Akhlaghian and colleagues [36] compared the marginal accuracy of zirconia copings fabricated using an intraoral scanner and three indirect scanning methods for different scanners. It was stated that marginal adaptation of all zirconia copings fabricated with four scanning techniques was within a clinically acceptable range. However, the best digitization method was the extraoral laboratory scanner, and the maximum vertical marginal gap was for intraoral scanning. Rajan and co-workers [37] evaluated the marginal adaptation of zirconia copings fabricated by two CAD-CAM systems (CERAMILL and CEREC -In Lab MC XL), CEREC -In Lab MC XL (68 μm) performed higher marginal discrepancies. Baig and colleagues [38] evaluated the marginal fit of Yttria-stabilized tetragonal zirconia polycrystals (Y-TZP) ceramic crowns compared to IPS Empress II and complete metal crowns fabricated using a digital system. The Cercon system (Y-TZP) demonstrated significantly higher marginal gaps than the other two systems.

## Conclusion

Within the limitations of this clinical study, monolithic zirconia FPDs fabricated from both scannable impression materials and conventional impression materials were not superior to each other in marginal accuracy by evaluating with the replica technique.

## Data Availability

The datasets generated during and analyzed during the current study are not publicly available since the manuscript has not been accepted yet, but are available from the corresponding author on reasonable request.
